# Enhancing health literacy and academic success through virtual reality in medical imaging education

**DOI:** 10.3389/fmed.2025.1703740

**Published:** 2025-12-22

**Authors:** Nuran Akyurt, Wiam Elshami, Huseyin Ozan Tekin

**Affiliations:** 1 Vocational School of Health Services, Marmara University, Istanbul, Türkiye; 2 College of Health Sciences, University of Sharjah, Sharjah, United Arab Emirates; 3 Computer Engineering Department, Faculty of Engineering and Natural Sciences, Istinye University, Istanbul, Türkiye

**Keywords:** virtual reality, immersive learning, experiential learning, student satisfaction, medical education, health literacy

## Abstract

**Introduction:**

This quasi-experimental study examined the effectiveness of integrating virtual reality (VR)-based training into the medical imaging curriculum, particularly in enhancing eHealth literacy, health-related behaviors, and academic outcomes.

**Methods:**

A total of 96 students participated, divided into a VR-based training group (*n = 35*) and a traditional training group (*n = 61*). Grounded in Kolb’s Experiential Learning Theory, Sweller’s Cognitive Load Theory, and the Cognitive-Affective Model of Immersive Learning (CAMIL), the study aimed to explore how immersive, student-centered learning environments influence health education outcomes. Data were collected using the eHealth Literacy Scale (eHEALS), Health-Related Behaviors Scale (HBS), Center for Epidemiological Studies Depression Scale (CES-D), and Breast Health and Examination (BHE) test in both groups. The VR-based group also completed the Student Satisfaction and Self-Confidence in Learning Scale (SCLS).

**Results:**

Post-training BHE scores increased significantly in both groups (*p* < 0.001), though no statistically significant difference was observed between the groups in eHEALS, HBS, CES-D, or BHE scores. The VR-based group reported high satisfaction and self-confidence (SCLS mean = 54.1 ± 4.1).

**Conclusion:**

The use of scenario-based virtual patients and simulated breast models created a reflective, low-risk, and student-centered learning environment aligned with experiential and cognitive learning theories. The VR-supported breast health curriculum was associated with comparable learning outcomes to the traditional course, suggesting that immersive methods can provide an alternative mode of delivery in health education to foster motivation, self-efficacy, and clinical competence.

## 1 Introduction

Virtual reality (VR) is an advanced technology used to create computer-generated environments that closely resemble the real-world environment or situation (1). In the health field, VR is used as an adjunct tool in practical skill training that enables students to practice in a very low-risk environment before facing real patients (2, 3).

Recent theoretical advances suggest that the integration of immersive technologies into education can be better understood through specific learning frameworks. This study was conceptually grounded in three complementary learning theories:

1. Kolb’s Experiential Learning Theory, which emphasizes learning through concrete experience, reflective observation, abstract conceptualization, and active experimentation (4);
2. Sweller’s Cognitive Load Theory, which highlights the importance of reducing extraneous load in instructional design to facilitate effective cognitive processing (5, 6); and
3. the Cognitive-Affective Model of Immersive Learning (CAMIL), which explains how technological factors (e.g., immersion, fidelity), psychological constructs (e.g., motivation, presence, agency), and emotional factors (e.g., self-efficacy, embodiment) interact to produce learning outcomes in immersive environments (7).

These frameworks collectively support the design and evaluation of VR-based interventions by addressing both cognitive processing and affective engagement in virtual environments.

Use of “scenario-based” virtual patient programs is considered to be effective to meet strategic rather than procedural learning goals (8). Hence, there is potential for VR to act as an effective tool to facilitate in depth learning of educational materials and the acquisition of clinical reasoning skills in the healthcare setting, through a safe learning environment with the opportunity for repetitive practice and real-time feedback (2, 8–10).

The integration of simulation and task-based scenarios aligns with the success of other serious game platforms, such as the Immersive Virtual Anatomy Laboratory (IVAL) (11), while studies also confirm that immersive technologies, including AR and VR, significantly improve spatial understanding and interactivity compared to traditional methods (12).

Clinical breast examination (CBE), as a key tool in the breast cancer screening for early detection of breast cancer (13), is considered an important clinical skill that should be taught as part of undergraduate medical curricula (9, 14). However, students often have limited exposure to intimate examinations such as pelvic, prostate, and breast examinations, and they consider CBE as an anxiety-provoking task necessitating an additional training (9, 14).

As an innovative way to augment traditional methods of clinical skill acquisition, the use of VR technology via simulated breast models and virtual patients can facilitate the learning CBE by improving the student comfort levels before interacting with a real patient (9, 14).

The concept of eHealth literacy has emerged as a result of the widespread use of electronic resources and the Internet for searching the health-related information with rapid development of information technologies (15, 16). The eHealth literacy is defined as the ability to search, obtain and appraise health information from electronic sources and apply the acquired information to address or solve a health problem (17).

eHealth literacy is considered a key area of the eHealth ecosystem, which varies among populations, groups, and job categories, and is considered essential for improving healthcare delivery and quality of care (15, 16, 18). Accordingly, students in the health field should have a critical perspective on health information and proficient eHealth literacy skills as an important resource for clinical and educational practice (15, 16, 18, 19).

University years are the most important period for acquiring lifelong positive health behavior, since health promoting behaviors can be easily adopted in this period, while a higher level of e-health literacy is suggested to facilitate this process (20–22). Although university students frequently use information technologies, the eHealth literacy level as well as its correlates are less extensively studied in this group, including those studying in the field of health (15, 20, 23–25).

Indeed, the health literacy is considered one of the key factors affecting learning, while the education programs tailored to literacy levels and learning style are associated with the most effective on outcomes (26). VR-based tools address the health literacy in several aspects by facilitating the health-related understanding and information application, with a potential to provide superior outcomes to conventional methods of health-related information delivery (10).

Therefore, this study aimed to investigate the utility of a VR-based training program, which was provided within the context of courses on breast health and examination in the curriculum of a medical imaging techniques program, via a comparative analysis with the traditional training in terms of eHealth literacy, health-related behaviors and academic success.

## 2 Materials and methods

### 2.1 Study design

The study was designed based on the integration of experiential and cognitive learning theories into virtual reality-supported education. Specifically, Kolb’s Experiential Learning Theory, Sweller’s Cognitive Load Theory, and the Cognitive-Affective Model of Immersive Learning (CAMIL) were adopted to guide the design and evaluation of the educational content and technology used (4–7).

### 2.2 Study population

**FIGURE 1:**
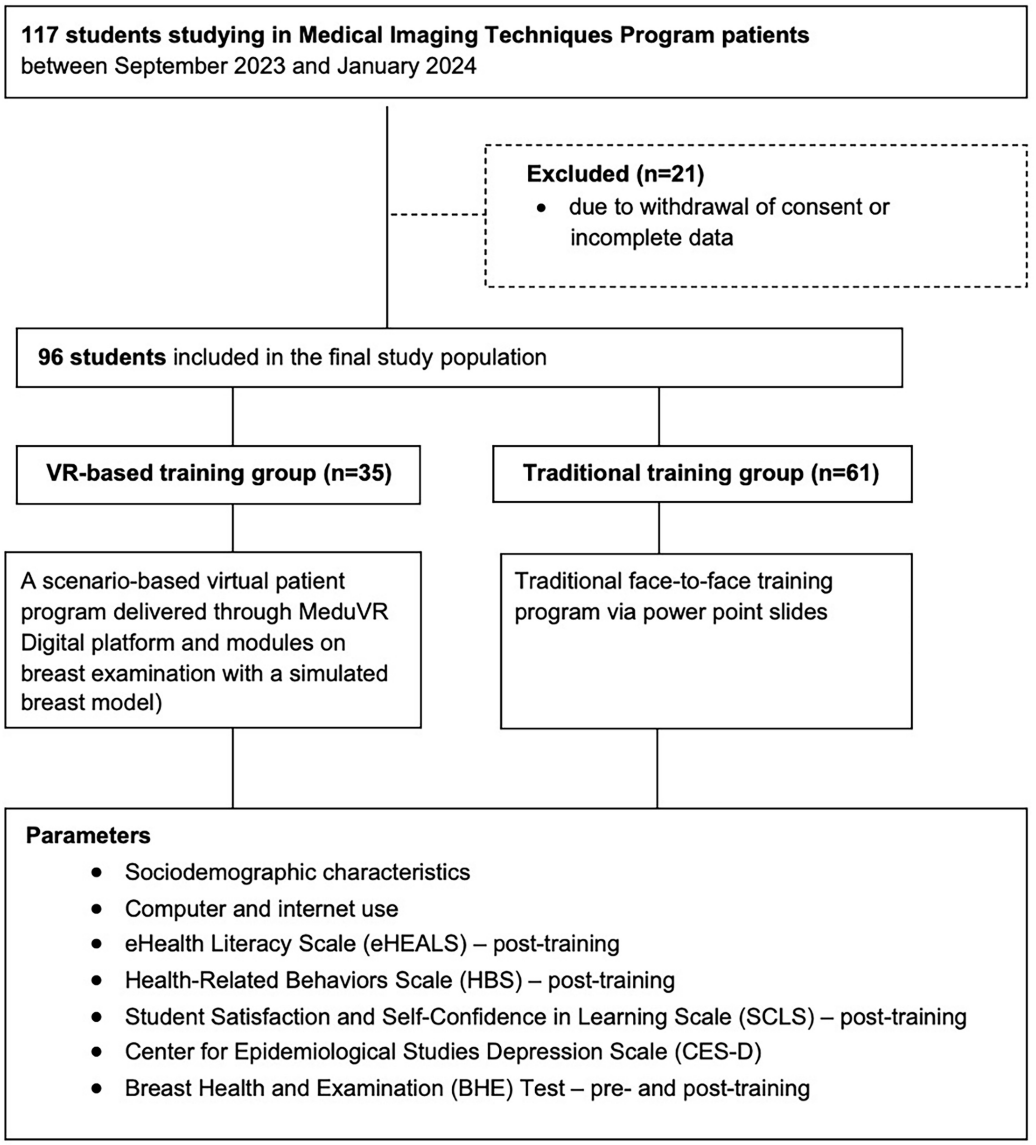
Study flowchart.

A total of 96 students studying in Medical Imaging Techniques Program of Marmara University Vocational School of Health Services were included in this quasi-experimental questionnaire-based study conducted between September 2023 and January 2024. From the total pool of eligible students enrolled in the Medical Imaging Techniques Program (*n* = 117), the final sample consisted of 96 participants with exclusion of 21 students due to withdrawal of consent and incomplete data. The participants were allocated to either VR-based training (*n* = 35; a scenario-based virtual patient program delivered through MeduVR Digital platform and modules on breast examination with a simulated breast model) or traditional training (*n* = 61; traditional face-to-face training program via power point slides) groups, based on the training program applied within the context of courses on breast health and examination in the curriculum (Figure 1). Students who withdrew or discontinued participation did not provide demographic or baseline information due to ethical restrictions; therefore, no additional attrition comparison could be performed beyond the available data.

Written informed consent was obtained from each participant. The study was conducted in accordance with the ethical principles stated in the “Declaration of Helsinki” and approved by Marmara University Clinical Research Ethics Committee (Date of approval: 08/09/2023, Protocol no: 09.2023.1111).

### 2.3 Assessments

The study questionnaire involved four parts including Part 1 – items on participants’ sociodemographic characteristics and the computer and internet use, Part II – items on eHealth Literacy Scale (eHEALS) and Health-Related Behaviors Scale (HBS), Part III – items on Student Satisfaction and Self-Confidence in Learning Scale (SCLS), and Part IV – items on the Center for Epidemiological Studies Depression Scale (CES-D). The study scales were applied in both groups after the training, while the SCLS was applied only in the VR-based training group. Also, each student received the 40-item “Breast Health and Examination (BHE) Test” (scored 0–100) both in the pre-training and in the post-training period to determine the efficacy of provided training (Figure 1).

### 2.4 Product design

The structure and content of the VR-based training program were informed by Kolb’s Experiential Learning Theory to promote active and reflective learning, and by Sweller’s Cognitive Load Theory to ensure efficient cognitive processing. Moreover, the immersive nature and user-centered design of the MeduVR platform were aligned with the constructs of the CAMIL model (presence, agency, embodiment, and self-efficacy) to foster engagement and deeper learning outcomes (4–7).

The VR-based training program was developed in accordance with the six-step approach for curriculum development for medical education, including problem identification and general needs assessment, targeted needs assessment, goals and objectives, educational strategies, implementation, evaluation and feedback.

MeduVR Digital platform^1^, as developed within the scope of the project called “The Development of a Virtual Reality Technology Based Breast Cancer Early Diagnosis And Screening Prototype - Medical Device Biomedical Equipment Technologies,” was used as a learning management system (LMS) to deliver the scenario-based virtual patient program.

The MeduVR Digital platform is an online learning platform using a VR technology (integrated to VR headset and 3D VR glasses) and a user-friendly interface to improve the learning experience via utilization of interactive 3D models and simulations, access to course content and sharing of assignments. LMS also has an integrated system with analytical instruments to manage, monitor and assess the scenarios as well as collection of students’ performance data to evaluate the effectiveness of the learning period and to perform necessary improvements. The program was developed in line with the scientific contribution and expert clinical opinions of the scientific commission members of the Breast Health Association (MEMEDER) and the scientific opinions and recommendations of a multidisciplinary team consisting of breast surgeons, breast radiologists and breast nurses.

### 2.5 Training programs

The training structure allowed students to progress through experiential, hands-on sessions that encouraged active experimentation and reflection (4), while minimizing extraneous cognitive load through intuitive interface and modular design (5, 6), all within an immersive and emotionally engaging learning environment as described in the CAMIL model (7).

In the traditional training group, the 14-week course was completed through in-class face-to-face lecture format based on power point presentation.

**FIGURE 2:**
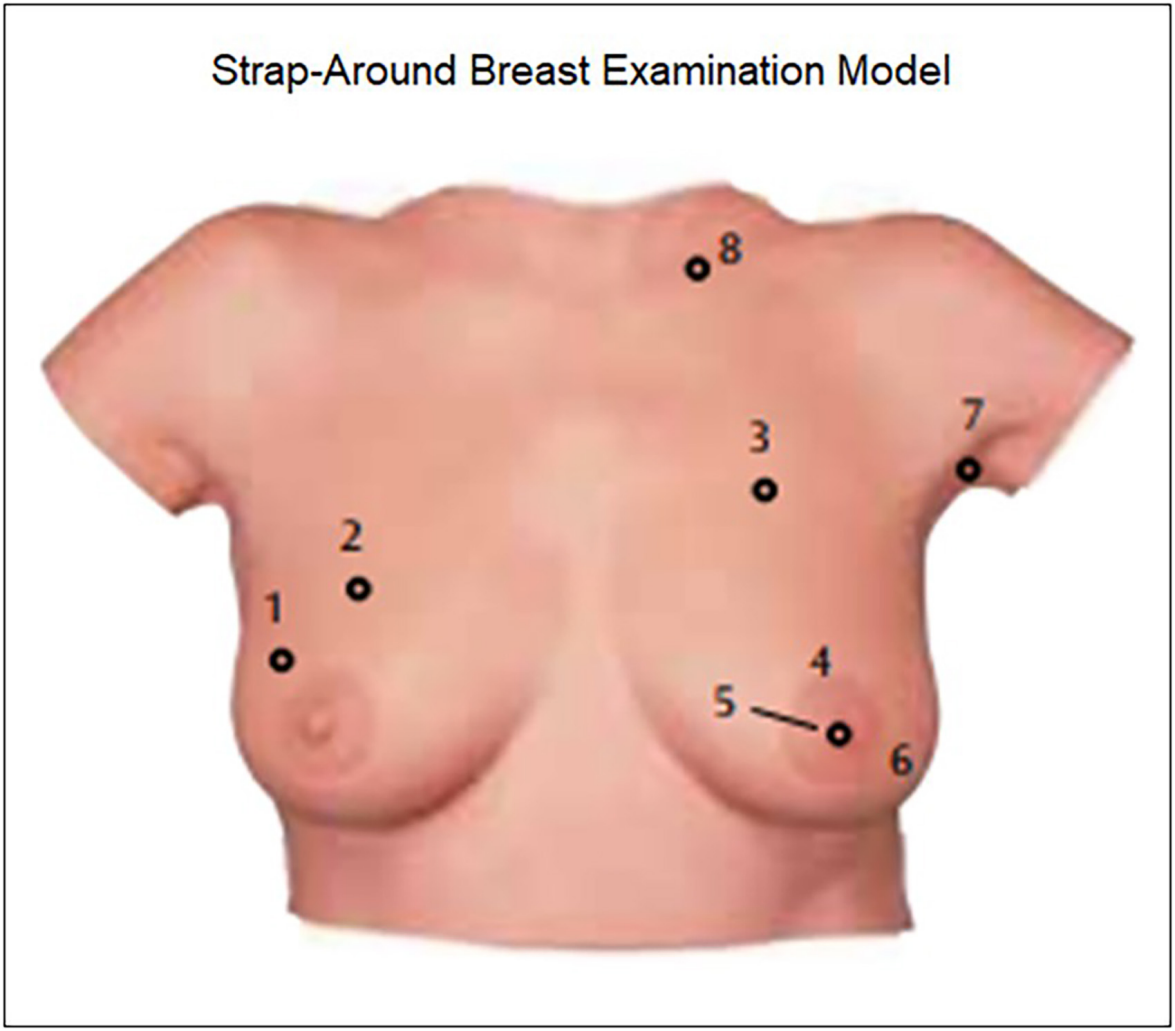
Strap-around breast examination model: a simulated breast model for clinical breast exam designed to provide tactile and real-time objective feedback which illustrates the eight different conditions: (1). Right breast: round, movable tumor at a depth of 10, 20 mm in diameter, presumably benign. (2). Right breast: round, movable tumor at a depth of 5, 20 mm in diameter, presumably benign. (3). Left breast: irregular tumor at a depth of 5 mm, adhering to the chest wall, diameter of approximately 35 mm × 25 mm, presumably malign. (4). Column-shaped, irregular tumor at a depth of 5 mm, adhering to the chest wall, approx. 30 mm in diameter, malign. (5). Left breast: permanently inverted nipple (recently occurred), frequently in combination with a malign tumor. (6). Left breast: “orange-peel skin,” skin structure as in an orange peel with pore retraction through lymphostasis caused by malign tumors. (7). Left armpit: irregular, firmly adhering lymph node at a depth of 10 mm, diameter approx. 35 mm × 25 mm, presumably malign. (8). Above left clavicle: Malign tumor at a depth of 5, 20 mm in diameter. (https://www.3bscientific.com/product-manual/L50_L51_L55_screen.pdf).

In the VR group, the training (2 h/week thought the 14-week course) was based on the scenario-based virtual patient program delivered through the MeduVR digital platform, a face-to-face course on breast health training and the applied course including modules on breast examination using the simulated breast model with implantable lesions (3B Scientific, Turkey) with the help of virtual reality (VR) headset and 3D VR glasses, and modules on clinical breast examination, breast self-exam and mammography prepared by the VR technology. Using Meta Quest 2 glasses, students performed palpation practice on plastic breast model while concomitantly visualizing 3D breast anatomy in VR environment. This was not an augmented reality (AR)-based system, and thus physical model and VR content were designed as parallel but separate experiences. The breast model consists of a natural real-size breast made of a dermatologically tested silicone (3B SKINlike*™* material, 3B Scientific, Turkey) enabling the feel and texture of a real breast with modules representing benign and malign (Figure 2).

### 2.6 The scenario-based virtual patient program

**FIGURE 3:**
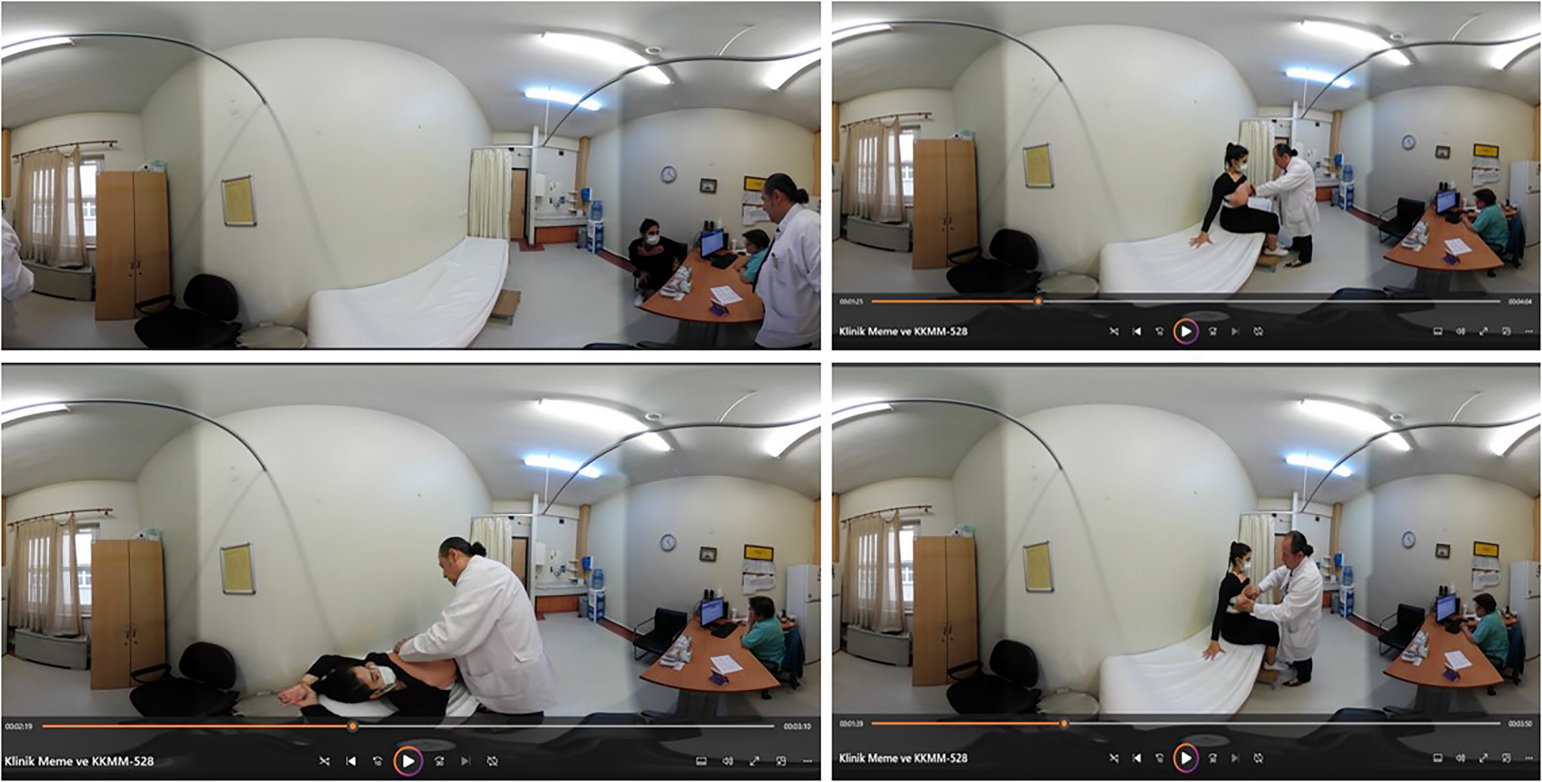
Digital content related to steps of clinical breast examination in a breast surgery outpatient clinic.

Virtual patient scenario provided data on stepwise evaluation of a patient (over 40 years of age) with a family history for breast cancer presenting to a breast outpatient clinic (patient characteristics, medical background, breast cancer risk factors, clinical breast examination, appropriate radiological imaging), which was followed by suggesting an individualized monitoring plan for each patient (Figure 3).

### 2.7 eHealth literacy scale (eHEALS)

The eHEALS is the most widely used tool for measuring eHealth literacy (19) which consists of eight items, each scored on a five-point Likert scale (from 1: strongly disagree to 5: strongly agree). The total score ranges from 8 to 40 with higher scores indicating higher eHealth literacy skills (27). Turkish validity and reliability of the scale was performed by Coskun and Bebis (28).

### 2.8 Health-Related Behaviors Scale (HBS)

Health-Related Behaviors Scale is a five-item scale assessing the behaviors to prevent disease and promote health, measured on a five-point Likert scale (1: strongly disagree to 5: strongly agree) (29).

### 2.9 Students’ Satisfaction and Self-Confidence Scale (SCLS)

Self-Confidence in Learning Scale is a 13-item scale used to measure student satisfaction with the simulation activity (5 items) and self-confidence in learning (8 items). The Turkish version consists of 12 items (5 items and 7 items for the satisfaction and self-confidence domains, respectively Each item is rated on a five-point Likert scale (1: strongly disagree to 5: strongly agree), with higher scores indicating higher satisfaction and greater levels of self-confidence (30). Turkish validity and reliability of the scale was performed by Karaçay and Kaya (31).

### 2.10 Center for Epidemiological Studies-Depression (CES-D)

Center for Epidemiological Studies-Depression is a 20-item measure assessing the frequency of experienced symptoms associated with depression (i.e., restless sleep, poor appetite, and feeling lonely). Each item is scored on a three-point Likert scale (from 0: rarely or never to 3: most or always), giving a total score of 0–60 with high scores indicating greater depressive symptoms. The CES-D also provides cutoff scores that aid in identifying individuals at risk for clinical depression (0–15: no depression; 16–20: mild depression; 21–30: moderate depression and ≥ 31: severe depression) (32, 33). Turkish validity and reliability of the scale was performed by Tatar and Saltukoğlu (34).

### 2.11 Statistical analysis

Using a convenience sampling in selecting participants from a population of total 125 students studying in the Medical Imaging Techniques Program, at least 94 participants were calculated to be included via sample size estimation based on a power of 90% at a type I error of 0.05.

Statistical analysis was made using IBM SPSS Statistics for Windows, version 24.0 (IBM Corp., Armonk, NY). Chi-square (χ2) test was used for the comparison of categorical data. Because the scale scores were derived from five-point Likert-type items and several subgroups showed deviations from normality according to the Shapiro–Wilk test and Q–Q plot inspection, non-parametric tests were used. Two-group comparisons were performed using the Mann–Whitney U test, whereas comparisons involving three or more groups were analyzed using the Kruskal–Wallis test followed by Bonferroni-adjusted pairwise Mann–Whitney U *post hoc* analyses when needed. Descriptive statistics were reported as mean ± SD, while all inferential *p*-values originate from non-parametric tests. Data were expressed as “mean ± standard deviation (SD),” minimum−maximum and percent (%) where appropriate. *p* < 0.05 was considered statistically significant.

## 3 Results

### 3.1 Sociodemographic characteristics and computer/internet use (*n* = 96)

Overall, 52.1% of students were in the 18–21 years age group, and females comprised the 68.8% of the study population (Table 1).

**TABLE 1:** Sociodemographic characteristics, computer and internet use (*n* = 96).

Parameters		
**Sociodemographic characteristics, n (%)**		
Age (year) Gender Training group Family type Maternal educational status Paternal educational status Monthly income	18–21 years	50 (52.1)
	22–25 years	46 (47.9)
	Male	30 (31.3)
	Female	66 (68.8)
	Traditional training	61 (63.5)
	Virtual reality-based training	35 (36.4)
	Nuclear	40 (41.7)
	Extended	32 (33.3)
	Shattered	24 (25.0)
	Illiterate	12 (12.5)
	Primary-secondary education	73 (76.0)
	Higher education	11 (11.4)
	Illiterate	7 (7.3)
	Primary-secondary education	67 (69.8)
	Higher education	22 (22.9)
	Low (below minimum wage)	64 (66.7)
	Moderate (minimum wage)	10 (10.4)
	High (above minimum wage)	22 (22.9)
**Computer and internet use, n (%)**		
Daily internet use Having a personal computer Computer skills Purpose of using ICT Looking for health information online (last week)	Less than 1 h	11 (11.4)
	1–3 h	42 (43.7)
	At least 4 h	43 (44.9)
	Yes	45 (46.8)
	No	51 (53.2)
	None	11 (11.5)
	Poor level	10 (10.4)
	Medium level	50 (52.1)
	Strong level	25 (26.0)
	Academic	15 (15.6)
	Communication (e-mail, chat, forum)	7 (7.3)
	Games and entertainment	71 (74.0)
	Social media applications	3 (3.1)
	1 h per day	16 (16.7)
	2–3 h per day	38 (39.6)
	1 h every 2 days	30 (31.2)
	1–2 h weekly	12 (12.5)

ICT, information and communication technology.

### 3.2 Post-training scale scores

**FIGURE 4:**
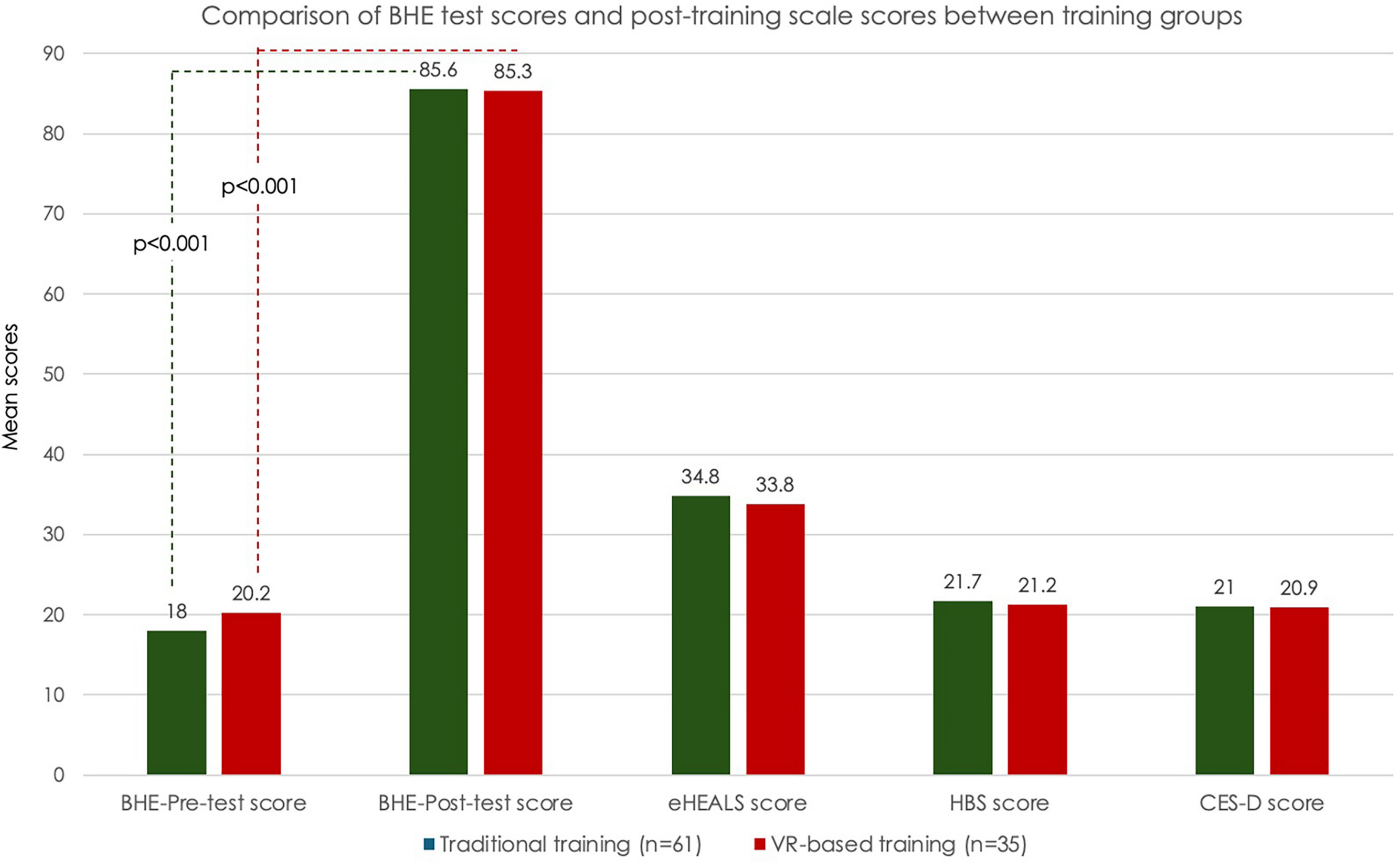
Pre-test and post-test scores on BHE (Breast Health and Examination (BHE), and the post-training eHEALS (eHealth Literacy Scale), HBS (Health-Related Behaviors Scale) and CES-D (Center for Epidemiological Studies-Depression) scores in training groups.

The mean ± SD scores of the study scales in the overall study population were found as follows: eHEALS (34.5 ± 4.3; Cronbach alpha: 0.983), HBS (21.5 ± 2.8; Cronbach alpha: 0.990) and CES-D (21.0 ± 5.5; Cronbach alpha: 0.628) (Figure 4 and Table 2).

**TABLE 2:** Post-training scale scores and Breast Health and Examination (BHE) pre-test and post-test scores.

Parameters	Traditional training (*n* = 61)	VR-based training (*n* = 35)	*P*-value
**Post-training scale scores, mean ± SD**			
eHEALS	34.8 ± 3.9	33.8 ± 4.8	0.228
HBS	21.7 ± 2.7	21.2 ± 3.0	0.409
CES-D	21.0 ± 5.6	20.9 ± 5.3	0.854
**CES-D category, *n* (%)**			
No depression(score 0–15)	9 (9.4)	4 (4.2)	0.900
Mild depression (score 16–20)	26 (27.1)	14 (14.6)	
Moderate depression (score 21–30)	20 (20.8)	14 (14.6)	
Severe depression (score ≥ 31)	6 (6.3)	3 (3.1)	
**BHE test scores, mean ± SD**			
Pre-test score	18.0 ± 6.0	20.2 ± 8.3	0.244
Post-test score	85.6 ± 3.0	85.3 ± 1.7	
Mean difference	67.5	65.1	–
95% CI for mean difference	66.3–68.7	63.2–67.2	–
Cohen’s d*	14.3	10.8	–
*P*-value (pre-test vs. post-test)	**<0.001**	**<0.001**	–

Values in bold indicate statistical significance (*p* < 0.05).

*Large d values for BHE are driven by the large pre–post difference and low post-test variability. Mean ± SD values are provided as descriptive statistics. All *p*-values were obtained using non-parametric tests (Mann–Whitney U for two-group comparisons; Kruskal–Wallis with Mann–Whitney U post-hoc tests for variables with more than two groups).

According to CES-D categories, mild depression and moderate depression was noted in 41.7% and 35.4% of students, respectively (Figure 4 and Table 2).

No significant difference was noted between the training groups in terms of eHEALS, HBS and CES-D scores (Figure 4 and Table 2).

### 3.3 BHE test scores

Overall, BHE post-test scores were significantly higher than BHE pre-test scores in the traditional training (85.6 ± 3.0 vs. 18.0 ± 6.0, *p* < 0.001), and VR-based training (85.3 ± 1.7 vs. 20.2 ± 8.3, *p* < 0.001), groups (Table 2).

Traditional (mean difference [95% CI]: 67.5 [66.3–68.7], Cohen’s d: 14.3) and VR-based (mean difference [95% CI]: 65.1 [63.2–67.2], Cohen’s d: 10.8) training groups demonstrated very strong within-group improvements in BHE performance. The exceptionally large Cohen’s d values for BHE are due to the large pre–post mean difference and the very small post-test variance, rather than a computational error (Table 2).

No significant difference was noted between training groups in terms of BHE pre-test and post-test scores (Figure 4 and Table 2).

### 3.4 Study scales and pre-test BHE scores with respect to sociodemographic characteristics and computer/internet use

The parental education level and amount of daily internet use were the only parameters with significant impact on scale scores and pretest scores (Table 3).

**TABLE 3:** Study scales and pre-test Breast Health and Examination (BHE) scores with respect to sociodemographic characteristics and computer/internet use.

	Scores, mean ± SD			
Parameters	eHEALS	HBS	CES-D	Pre-test BHE
**Age groups**				
18–21 years	34.9 ± 3.8	21.9 ± 2.6	21.0 ± 5.5	17.4 ± 6.2
22–25 years	34.0 ± 4.7	21.1 ± 2.9	20.8 ± 5.5	20.4 ± 7.5
*P*-value	0.380	0.261	0.944	**0.025**
**Gender**				
Male	34.1 ± 4.7	21.3 ± 2.9	20.3 ± 5.3	19.2 ± 9.2
Female	34.64.1	21.6 ± 2.7	21.3 ± 5.6	18.7 ± 5.8
*bP*-value	0.496	0.582	0.444	0.425
**Maternal education**				
Illiterate	37.2 ± 3.5	23.1 ± 2.4	24.4 ± 4.5	14.12 ± 2.7
Primary-secondary education	34.3 ± 4.4*	21.4 ± 2.9*	20.5 ± 5.7	19.9 ± 7.5**
Higher education	32.7 ± 2.4**	20.5 ± 1.5**	20.2 ± 3.4	16.9 ± 4.0
*P*-value	**0.016**	**0.019**	0.063	**0.021**
**Paternal education**				
Illiterate	37.7 ± 3.9	23.6 ± 2.4	23.3 ± 4.3	13.6 ± 2.1
Primary-secondary education	34.2 ± 4.4	21.3 ± 2.9	20.5 ± 5.5	18.0 ± 5.5
Higher education	34.4 ± 3.6	21.6 ± 2.4	21.5 ± 5.7	22.9 ± 9.7**
*P*-value	0.112	0.129	0.38	**0.02**
**Monthly income**				
Low (below minimum wage)	36.4 ± 3.5	22.6 ± 2.4	21.4 ± 7.6	17.7 ± 4.5
Moderate (minimum wage)	32.6 ± 4.5	20.7 ± 3.2	21.1 ± 4.9	21.1 ± 10.4
High (above minimum wage)	34.8 ± 4.1	21.7 ± 2.7	20.8 ± 5.5	18.3 ± 5.7
*P*-value	0.065	0.237	0.981	0.618
**Daily internet use**				
Less than 1 h	35.3 ± 3.6	21.8 ± 2.4	24 ± 4.04	14.5 ± 4.0
1–3 h	33.8 ± 4.7	21.0 ± 2.9	19.5 ± 4.6	21.1 ± 8.6^q^
At least 4 h	34.4 ± 3.9	21.7 ± 2.7	21.8 ± 6.2	16.3 ± 3.4
*P*-value	0.346	0.444	0.066	**0.011**
**Having a personal computer**				
Yes	34.3 ± 4.7	21.6 ± 3.1	21.93 ± 6.5	18.1 ± 5.9
No	34.6 ± 3.9	21.5 ± 2.5	20.1 ± 4.4	19.5 ± 7.8
*P*-value	0.901	0.626	0.099	0.509
**Purpose of using ICT**				
Academic	35.9 ± 4.8	22.4 ± 3.2	21.9 ± 5.3	17.5 ± 5.8
Games and entertainment	34.2 ± 4.3	21.3 ± 2.7	19.1 ± 4.3	19.3 ± 7.3
Communication (e-mail, chat)	32.6 ± 1.5	20.7 ± 1.9	20.9 ± 5.8	19.3 ± 6.8
Social media applications	38.7 ± 2.1	24.7 ± 0.6	20.7 ± 1.2	13.1 ± 3.7
*P*-value	0.087	0.168	0.748	0.161
**Looking for health information online (last week)**				
1 h per day	33.5 ± 6.5	20.8 ± 4	20.81 ± 4.5	17.2 ± 5.3
2–3 h per day	34.3 ± 3.8	21.4 ± 2.6	21.0 ± 5.7	19.8 ± 8.6
1 h every 2 days	34.3 ± 3.4	21.4 ± 2.2	21.4 ± 5.8	16.9 ± 4.7
1–2 h weekly	36.8 ± 3.6	23.3 ± 2.3	19.7 ± 5.9	22.4 ± 6.7
*P*-value	0.168	0.102	0.83	0.275

Values in bold indicate statistical significance (*p* < 0.05). eHEALS, eHealth literacy scale; HBS, Health-Related Behaviors Scale; CES-D, Center for Epidemiological Studies-Depression; BHE, Breast Health and Examination. Mean ± SD values are provided as descriptive statistics. All *p*-values were obtained using non-parametric tests (Mann–Whitney U for two-group comparisons; Kruskal–Wallis with Mann–Whitney U post-hoc tests for variables with more than two groups).

*P < 0.05 and **p < 0.01 compared to illiterate mothers or fathers.

*^Q^p* < 0.05 compared to at least 4 h of internet use daily.

Students with illiterate mothers, as compared to those with mothers having primary/secondary education and higher education, had significantly higher eHEALS scores (37.2 ± 3.5 vs. 34.3 ± 4.4 and 32.7 ± 2.4, *p* = 0.034 and *p* = 0.004, respectively) and HBS scores (23.1 ± 2.4 vs. 21.4 ± 2.9 and 20.5 ± 1.5, *p* = 0.030 and *p* = 0.003, respectively) (Table 3).

Breast Health and Examination pretest scores were significantly lower in students with illiterate mothers (14.12 ± 2.7 vs. 19.9 ± 7.5, *p* = 0.009) and fathers (13.6 ± 2.1 vs. 22.9 ± 9.7, *p* = 0.004) vs. those with mothers and fathers having higher educational level (Table 3).

Students with at least 4 h of daily internet use had significantly lower BHE pre-test scores compared to those with 1–3 h of daily internet use (16.3 ± 3.4 vs. 21.1 ± 8.6, *p* = 0.014) (Table 3).

### 3.5 SCLS scores in the VR-based training group

In the VR-based training group, total SCLS scores were 54.1 ± 4.1 (Cronbach’s alpha: 0.892; 0.841 for satisfaction and 0.773 for self-confidence domains) (Table 4).

**TABLE 4:** SCLS scores in the virtual reality (VR)-based training group.

Parameters	SCLS scores, mean ± SD
Total score	54.1 ± 4.1
**Age groups**	
18–21 years	52.5 ± 3.9
22–25 years	53.2 ± 5.6
*P*-value	0.278
**Gender**	
Male	51.7 ± 5.9
Female	53.9 ± 3.9
*P*-value	0.253
**Maternal education**	
Illiterate	51.2 ± 4.4
Primary-secondary education	54.1 ± 3.8
Higher education	47.5 ± 8.7
*P*-value	0.146
**Paternal education**	
Illiterate	49.3 ± 0.5
Primary-secondary education	54.2 ± 4.1
Higher education	51.7 ± 6.6
*P*-value	0.084
**Monthly income**	
Low (below minimum wage)	49.0 ± 0.0
Moderate (minimum wage)	54.0 ± 3.1
High (above minimum wage)	52.8 ± 5.5
*P*-value	0.525
**Daily internet use**	
Less than 1 h	55.5 ± 4.4*
1–3 h	53.7 ± 4.1*
At least 4 h	48.8 ± 5.2
*P*-value	**0.035**
**Having a personal computer**	
Yes	52.7 ± 4.1
No	53.2 ± 5.9
*P*-value	0.56
**Purpose of using ICT**	
Academic	55.5 ± 1.0
Games and entertainment	52.6 ± 5.3
Communication (e-mail, chat)	54.0 ± 0.0
Social media applications	–
*P*-value	0.67
**Looking for health information online (last week)**	
1 h per day	50.5 ± 2.7
2–3 h per day	55.5 ± 3.4
1 h every 2 days	52.3 ± 6.7
1–2 h weekly	52.8 ± 4.4
*P*-value	0.148

Value in bold indicates statistical significance (*p* < 0.05). SCLS, Student Satisfaction and Self-Confidence in Learning Scale. Mean ± SD values are provided as descriptive statistics. All *p*-values were obtained using non-parametric tests (Mann–Whitney U for two-group comparisons; Kruskal–Wallis with Mann–Whitney U post-hoc tests for variables with more than two groups).

*P < 0.05 compared to at least 4 h of internet use daily.

Self-confidence scores were similar across the subgroups of sociodemographic characteristics. The daily internet use was the only parameter with significant impact on SCLS scores. Students with at least 4 h of daily internet use had significantly lower SCLS scores than those with less than 1 and 1–3 h of daily internet use (48.8 ± 5.2 vs. 55.5 ± 4.4 and 53.7 ± 4.1, *p* = 0.042 and *p* = 0.025, respectively) (Table 4).

## 4 Discussion

Our findings revealed that VR training yielded comparable outcomes to traditional education in terms of high levels of eHealth literacy and health promoting behaviors in students as well as the significantly improved knowledge scores after the training. Also, the SCLS scores in the VR-based training group were consistent with a high student satisfaction with the simulation activity and the self-confidence in learning.

These outcomes can be interpreted in light of Kolb’s Experiential Learning Theory, which emphasizes that learning is most effective when students actively engage in concrete experiences and reflect upon them. The VR-based modules in our study allowed students to perform virtual clinical breast examinations in a simulated, low-risk environment—an experience that aligns with Kolb’s model by facilitating learning through action, observation, and reflection.

Furthermore, the instructional design of the VR platform and materials was intentionally structured to align with Sweller’s Cognitive Load Theory, which highlights the importance of minimizing extraneous cognitive load while optimizing intrinsic and germane load (6, 7). The intuitive interface of the MeduVR platform, the scenario-based linear patient journey, and visual navigation tools all aimed to reduce unnecessary cognitive effort, allowing learners to focus more deeply on the intended learning objectives and decision-making processes.

In addition, the immersive nature of the training program is conceptually supported by the CAMIL, which explains how factors such as presence, agency, and emotional engagement enhance the learning process. Students in the VR-based training group demonstrated high satisfaction and self-confidence scores (SCLS), which can be interpreted as an outcome of increased psychological presence and perceived self-efficacy—two core components of the CAMIL model. The ability to make clinical decisions in a simulated space likely fostered a sense of autonomy and ownership over the learning experience (7).

Taken together, these theoretical perspectives offer a holistic explanation for the effectiveness of the VR-based intervention. By combining experiential engagement (4), efficient cognitive structuring (5, 6), and immersive emotional involvement (CAMIL model) (7), the educational intervention appears to support both cognitive and affective domains of learning. This integrated approach is especially valuable in clinical skills training, where learner anxiety and confidence are known to influence skill acquisition. Similarly, many studies and systematic reviews regarding the applications of VR-based training in the setting of health education indicated that VR applications are at least as effective as traditional learning methods in improving the knowledge levels and decision-making skills of students (2, 35–37).

The VR-based learning is considered to have augmented effects in terms of improved self-confidence of learners and creation of an attractive learning environment compared to conventional learning approaches (2, 35–37). Accordingly, the high scores for student satisfaction with the simulation activity and self-confidence in learning in our VR-based training group indicates the successful integration of the VR technology to the training program in this regard, with utilization of the scenario-based virtual patient program and the simulated breast model for CBE. Likewise, a single interaction with a virtual patient with a breast complaint and breast mannequin was reported to be associated with improved self-confidence of students in breast examination during a surgery clerkship (9). These findings are consistent with immersive learning research, such as the IVAL project, which similarly demonstrated increased satisfaction and efficiency among health students using VR-based anatomy training (38).

In alignment with the theoretical assumptions of the CAMIL model (7) 2021, the present study also examined the associations between student satisfaction, self-confidence, and learning performance. Although the correlations between SCLS and BHE post-test scores were positive yet weak and non-significant (*r* = 0.103–0.108, *p* > 0.30), the direction of these relationships supports the conceptual premise that satisfaction and confidence contribute indirectly to learning. Rather than directly influencing performance, these affective components are likely to enhance engagement, motivation, and perceived relevance of the learning experience. This interpretation aligns with prior research showing that immersive and interactive learning environments primarily promote attitudinal and motivational gains, which subsequently facilitate cognitive outcomes (7, 39–41).

Nonetheless, our findings revealed that the VR intervention was not statistically superior to traditional training in the primary objective measures (BHE, eHEALS, HBS), and thus the immersive, experiential VR training was only equally effective to the traditional group. The high mean BHE score post-intervention in both groups suggests the assessment may have been too easy, resulting in a ceiling effect that masked any potential significant difference between the two methods. This pattern also explains the unusually large Cohen’s d values, which reflect the strong learning effect and the narrow distribution of post-test scores. However, very low pre-test scores demonstrate substantial knowledge gain, indicating that the test was not inherently too easy. Besides the potential ceiling effect of the BHE test, the limited 2-h/week intervention duration, or the high quality and inherent effectiveness of the 14-week traditional course may be considered as other possible explanations for the non-superiority Also, short-term VR interventions, despite high user acceptance, may not translate into behavioral modification such as stronger positive health behaviors or improved online health information appraisal skills. Accordingly, longer interventions supported with behavioral feedback modules seems to be necessary to significantly boost eHealth literacy (eHEALS) or Health-Related Behaviors (HBS).

In addition, as the study employed a quasi-experimental design without random assignment and included post-only measures for several variables, the results should be interpreted as indicative of associations rather than causal effects. Thus, while the VR-based intervention appeared comparable to traditional training in academic outcomes, causality cannot be inferred.

Nonetheless, while the eHEALS, HBS, and CES-D scores were collected post-training only, these scores did not differ significantly across a wide range of sociodemographic and digital-technology–related variables, providing indirect evidence that substantial pre-intervention differences between groups are unlikely. Also, SCLS scores within the VR-based group did not vary significantly across the same sociodemographic factors, indicating that students’ satisfaction and self-confidence in learning were consistent regardless of demographic or digital background. This internal consistency supports the notion that the VR group represented a relatively homogeneous student profile, further reflecting the uniformity of the overall sample. Taken together, these findings do not eliminate the methodological limitation of having post-only measurements for eHEALS, HBS, and CES-D; however, they do suggest that the study sample possessed broadly comparable sociodemographic and digital characteristics. This homogeneity provides some contextual support for the interpretability of post-training comparisons.

In previous studies among university students in Turkey, the eHEALS scores was reported to range from 28.7 to 29.4 in students studying in Vocational School of Health Services (15, 42), and to be similar in the health and non-health fields (20, 23). The eHEALS scores of Canadian pharmacy students were reported to be 31.0 ± 4.3 (43), while Taiwanese nursing students had eHEALS scores of 31.4 ± 4.4 (24). In our cohort, the e-HEALS scores (34.5 ± 4.3) were consistent with high e-Health literacy levels, supporting the reinforcing effect of health-related education on the eHealth literacy, as the improved academic experience and knowledge affect the eHealth literacy skills in a positive manner (15, 35).

The adequate health literacy among healthcare providers is considered a vital prerequisite for the successful adoption of emerging technologies in healthcare delivery and thus provision of superior healthcare services (20, 23, 44). Indeed, VR may act as a facilitator for improved health literacy, further supporting the recommendations on incorporating VR simulation in curriculum and classes of health care professional programs (9, 45).

Health literacy is suggested to be positively associated with self-care and quality of life and to be negatively associated with functionality, mental health and depressive symptoms (46–48). Hence, the concomitantly high scores on e-HEALS and HBS in our students support that having a higher level of e-health literacy enables individuals to adopt more than one behavior that is positive for their health (20–22). The high eHEALS scores in our student is also consistent with their concomitantly low depression scores, given the reported relationship between poor health literacy and increased risk of having symptoms of depression (24, 46–48).

In our study, age, gender or income level had no significant impact on students’ eHEALS scores, which seems consistent with previous studies indicating no significant relationship of e-health literacy with the age, gender or income level (15, 16, 25, 49, 50). However, some studies indicated higher eHEALS scores in females (22), in males (19, 24), in students aged > 24 years (42) and in students with higher income levels (15, 51).

Importantly, lower maternal educational level was associated with increase in the eHEALS scores and HBS scores in our students, supporting the data from previous studies regarding the higher eHEALS scores in students whose mothers were primary school graduates (15, 49). The significant impact of maternal education on the e-health literacy levels of children seems notable given the critical value of the women’s e-Health literacy, as a person who forms the basis of the family, in terms of public health promotion strategies (15). Nonetheless, some studies in adolescents reported higher eHEALS scores with higher maternal educational level in (52, 53), while no relationship between maternal education level and eHEALS scores was found in a study with nursing students (39). Consistent with previous studies, our findings revealed no significant impact of the father’s education level on students’ eHEALS scores (39, 52).

Nearly half of our students reported at least 4 hours of internet use daily, which seems in line with the growing increase in the time spent in the internet and social media from 2–3 to 4–6 h, particularly after the COVID-19 pandemic (20, 49). In our cohort, daily internet use had no significant impact on e-HEALS and HBS scores, supporting the previous studies indicated that eHEALS scores were not changed according to the frequency of internet use (15, 25, 42, 49). In fact, the association of an at least 4 h of daily internet use with lower scores on BHE pretest and lower SCLS scores, rather than affecting the e-HEALS and HBS scores, seems to be consistent with the use of internet for the purpose of online games and entertainment by majority of our participants. Notably, albeit not significant, both the e-HEALS and HBS scores were higher in our students using the internet mainly for the academic purposes.

Subjects with higher eHealth literacy are considered more likely to seek health information online (16, 54, 55). However, our findings revealed a non-significant tendency for higher e-HEALS and HBS scores in students with less frequent searching for online health information. The concomitantly high levels for the eHealth literacy and the health-related behaviors in our students supports the correlation between the self-perceived good health status and higher eHEALS scores (15, 56, 57). The individual with anxiety about their health and a low level of eHealth literacy are considered vulnerable to develop problematic search behavior online and to be at risk for maladaptive health-related behaviors (55, 58).

Notably, given the uncontrollable quality and credibility of online information, it has been emphasized that having a high eHEALS level does not guarantee having skills in assessing, differentiating between high- and low-quality health resources and appropriate use of online health information to address health problems (16, 24, 55). Accordingly, several health professional organizations recommend that health care professional programs include health literacy in their professional curricula to improve students’ eHEALS skills in terms of using online health information effectively by reaching the credible online resources (2, 16, 24, 35, 59, 60).

Although the current findings demonstrate significant short-term improvements in knowledge and self-confidence, the long-term pedagogical sustainability of immersive learning remains to be explored. Future longitudinal studies should examine whether repeated exposure to immersive environments fosters lasting motivation, retention, and skill transfer in clinical practice.

Certain limitations to this study should be considered. First, the qualitative cross-sectional study design limits the ability to make causal inferences. Second, the potential lack of generalizability seems another important limitation due to small samples size. Third, there are also limitations inherent to convenience sampling along with absence of random allocation and blinding, such as the possibility of selection bias and a lack of diversity leading to limited external validity. The results may reflect the characteristics of the program’s curriculum design and cultural context, including students’ attitudes toward breast health education and technology-enhanced learning. To enhance generalizability, future studies should replicate this VR-supported intervention across multiple institutions and in different cultural and disciplinary settings. Fourth, demographic data could not be collected from students who withdrew or provided incomplete data, which prevented a full attrition analysis. However, the final sample remained balanced across key variables (age, gender, and group allocation). Fifth, although prior VR experience was not directly measured, participants’ general technological exposure appeared balanced between groups. Sixth, the current study was a pilot phase lacking a follow-up assessment, which is another limitation in terms of claims about long-term knowledge retention, which is the true measure of a successful educational tool. Seventh, high post-test scores may indicate a partial ceiling effect that could have limited the discrimination between groups. Future studies should consider revising test difficulty levels or incorporating performance-based OSCE assessments to minimize ceiling saturation. In addition, very high Cronbach’s alpha coefficients (above 0.95) were observed for the eHEALS and HBS scales, which may indicate potential redundancy among items due to the high homogeneity of the study population. Future research should consider item-level analyses or short-form adaptations to verify construct robustness.

In conclusion, our study revealed that the integration of virtual reality (VR) into health education—specifically breast health training—resulted in high levels of eHealth literacy, positive health-related behaviors, and comparable academic success relative to traditional training methods. These results can be better understood and justified within the framework of established learning theories. Kolb’s Experiential Learning Theory supports the VR experience as a dynamic platform for concrete experiences and reflection. Sweller’s Cognitive Load Theory explains how the instructional structure helps learners process information efficiently. Finally, the CAMIL model illustrates the affective engagement and motivational mechanisms triggered by immersive learning, contributing to enhanced learner confidence and satisfaction.

The high satisfaction with the simulation activity and self-confidence in learning in the VR-based training group indicates the successful integration of the VR technology to the training program with utilization of the scenario-based virtual patient program and the simulated breast model for CBE. Our findings indicate the potential correlation between self-perceived good health status and higher eHealth literacy, while also emphasize the likelihood of content and purpose of internet use rather than its frequency in contributing to acquisition of proper eHealth literacy skills and health-related behaviors. To ensure sustainability of learning outcomes, future work should adopt longitudinal follow-up methods similar to C-IVAL (61). Additionally, future versions of MeduVR could incorporate AI-powered virtual tutors as in GenAiVR-Lab (62), and expand toward collaborative social VR learning environments guided by conversational AI, as suggested in recent Social VR classroom studies (63). Accordingly, besides the VR-based training, the inclusion of the eHealth literacy in the curriculum of medical imaging techniques program seems necessary to improve students’ eHEALS skills in terms of using online health information effectively by reaching the credible online resources.

## Data Availability

The raw data supporting the conclusions of this article will be made available by the authors, without undue reservation.

## Footnotes

1
https://meduvr.com/
